# Home environment factors associated with early childhood development in rural areas of Bangladesh: evidence from a national survey

**DOI:** 10.3389/fpubh.2023.1209068

**Published:** 2023-06-28

**Authors:** Farzana Rahman, Samiha Nahar Tuli, Prasenjit Mondal, Shakina Sultana, Asmita Hossain, Satyajit Kundu, Afrin Ahmed Clara, Ahmed Hossain

**Affiliations:** ^1^Department of Public Health, North South University, Dhaka, Bangladesh; ^2^Armed Forces Medical College, Dhaka, Bangladesh; ^3^School of Medicine and Dentistry, Griffith University, QLD, Australia; ^4^Z.H. Sikder Women’s Medical College and Hospital, Dhaka, Bangladesh; ^5^School of Public Health, Southeast University, Nanjing, China; ^6^Faculty of Nutrition and Food Science, Patuakhali Science and Technology University, Patuakhali, Bangladesh; ^7^College of Health Sciences, University of Sharjah, Sharjah, United Arab Emirates; ^8^Global Health Institute, North South University, Dhaka, Bangladesh

**Keywords:** early childhood development, delayed development, home environment, rural children, Bangladesh

## Abstract

**Background:**

Knowing the relationship between the factors related to home environment and early childhood development (ECD) in Bangladeshi children aged 3 to 4  years would help to find out appropriate interventions for the children with lower ECD outcomes. Therefore, we aimed to understand the relationship between the home environment factors and ECD in rural Bangladeshi children aged 3 to 4  years.

**Methods:**

We used data from the Multiple Indicator Cluster Survey (MICS) 2019, and included 7,326 rural children aged 3 to 4  years. The ECD index (ECDI) included four domains: literacy-numeracy, learning, physical and socio-emotional development. If a child met at least three of these four domains, the child was indicated as developmentally “on track”.

**Results:**

The findings show that 27.4% of rural children missed to reach developmentally on-track while 72.2% of children did not attain the literacy-numeracy domain of ECD. The home environment factors including parental participation in children’s activities, was found to be associated with ECD. For instance, reading books to child had 26% (aOR = 1.26, 95% CI = 1.08–1.48), and telling stories to child had 29% (aOR = 1.29, 95% CI = 1.09–1.53) more developmentally on-track in overall ECDI. Similar associations between home environment factors and specific ECD domains were also obtained. We also identified that children aged 4  years, girls, and children of mothers with higher socio-economic status (SES) were higher developmentally on-track than their counterparts.

**Conclusion:**

Home environment factors like reading books and telling stories to children were found to be significantly associated with ECD in rural areas of Bangladesh. Our study’s findings would assist in implementing the essential public health intervention to enhance the ECD program especially in the rural Bangladeshi context.

## Background

Early childhood development (ECD) is a process of growth and interaction, more specifically, it refers to the children’s cognitive, motor and social–emotional development (1). It has a substantial impact on well-being, competence in literacy and numeracy, as well as economic status throughout the lifespan of a child (2). A good foundation in the early years makes significant changes in all aspects of adulthood, facilitating a child’s contribution to financial and social welfare in the later stages of life (2). However, it is now estimated that 250 million children under five are not reaching their developmental potential in low-and-middle-income countries (LMICs) (3). Previous studies focusing on LMICs also revealed that three to four-year-old children lag behind in conquering their adequate learning and socio-emotional developmental status (2, 3). Given the significance, the United Nations has included ECD in target 4.2 of Sustainable Development Goals (SDG) to ensure access to care and pre-primary education for every child along with quality ECD for all girls and boys who are developmentally on tract in health, learning, and psychosocial well-being (4).

Childhood development related to poor socioeconomic status adversely affects brain development resulting in an inappropriate cognitive development (1). In the early stage of life, exposure to different risk factors of ECD immensely affects the course of life, ranging from poor school performance to economic constrain in adulthood (5). As this evidence is more comprehensible among the children residing in poor communities, their growth and survival need to be more essentially contemplated (2). Multiple biological, environmental, and psychological factors are responsible for the development of under-five children. Among them, multiple anthropometric failures, especially stunting and extreme poverty, are the persistent factors accelerating delayed ECD status (1). The home environment is evidenced to be associated with the ECD, which is defined as the emotional warmth parents show while interacting with their children, the availability of engaging learning opportunities within the house, and the physical surroundings such as play places and cleanliness (6). In this study, we emphasized the parents’ book reading, telling stories, singing songs to the children, and playful activities with children as home environment factors. The ideal childhood environment may help a child’s growth and has a consistent impact on the child’s cognitive capacity and long-term personal potential (5, 7–9). Previous study shows that a child’s growth can be influenced, mediated, and regulated by a stimulating and high-quality home environment (10). Furthermore, childhood experiences in a good environment setting can help children not just achieve their intellectual potential later in life, but also build personal endurance (7, 11). The consideration of issues related to child protection such as child labor and school dropout, is imperative in addressing the development of children (12). At the same time, a child’s growth and conduct might be harmed by a poor home environment (13). According to UNICEF, children’s development is influenced by the environment in which they grow up and their interactions with their parents and caregivers. Because rapid brain growth happens at this time of life, caregivers’ involvement in children’s learning activities improves numerous elements of brain development, such as physical, social, cognitive, and emotional uplift (14, 15). Therefore, this study emphasizes the importance of the children’s home environment factors on their early childhood development status.

Potential risk factors for delayed development also include maternal physical and mental health conditions and maternal malnutrition (16). Nevertheless, maternal education, breastfeeding, and family interaction are some of the protective factors to ensure adequate ECD status (16). Also, parents’ involvement in various learning activities of children has been documented as the strongest predictor of cognitive as well as overall development of children (17, 18). Parents’ book reading significantly influenced children’s literacy skills (18). Parent-children’s playful activities were also an attributable factor for positive child development (18). In addition to parent–child interaction, socio-economic status also plays a significant role in child development. Impoverished wealth creates scarcity of food, sanitation, and proper hygiene, resulting in impeded child development due to economic strain, especially in rural areas where opportunities for children are minimal (2, 16). Lack of maternal education detrimentally affects children’s literacy, numeracy, and overall development (19). Additionally, adequate and proper nutrition is the major prerequisite during these crucial years of life since suboptimal nutrition can have a detrimental effect on the brain and all other domains of development (5). This nutritional deficiency can have deleterious effects on both short- and long-term cognitive and academic performances (20, 21). The phenomenon of overcrowding at home has the potential to impact both the health and development of children (22).

The Lancet 2016 ECD Series reported that 43% of children under five fail to achieve their developmental potential each year (23). Bangladesh’s ECD condition is not at all encouraging, like that of many other developing countries. A WHO assessment identifies Bangladesh as one of the ten countries with the most disadvantaged children who are at the greatest risk of having their cognitive and social–emotional development severely hampered (24). Moreover, the nutritional status and other health indicators of children and socioeconomic status vary between the urban and rural areas, with the lagging situation is observed in the rural settings in Bangladesh. However, nationally representative empirical research on ECD status among the rural children in Bangladesh is lacking. Despite the fact that ECD is an integral part of health policy in the majority of developed nations, there is no indication that Bangladesh pays adequate attention to this topic. The parenting techniques employed by rural Bangladeshi households are inadequately comprehended, and the socio-economic disparities in rural areas may impede the progress of child development (25). According to a report by UNICEF, the majority of rural mothers were uneducated, and nearly half of them did not understand the value of encouraging a child’s curiosity and self-confidence (26). Besides, the variations in the treatment of children and home environments in urban and rural settings particularly raise an important question.

Therefore, given the importance of the current ECD situations in children from rural Bangladesh, this study aimed to investigate the association between home environment factors and ECD status among the rural children of Bangladesh. The findings of this study could be crucial for providing important guidance to policymakers for formulating effective and timely interventions to improve ECD attainment in the rural context of the country.

## Methods

### Data source

**Figure 1:**
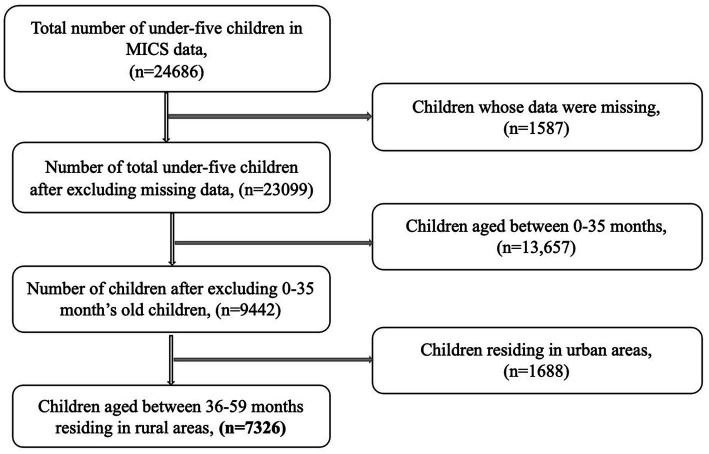
Flow diagram of selecting samples for the analysis from MICS data.

This secondary analysis utilized data from the Multiple Indicator Cluster Survey (MICS), a nationwide survey conducted in 2019 as a part of the global MICS program by the Bangladesh Bureau of Statistics in collaboration with UNICEF Bangladesh. To represent nationally representative and statistically significant data, 64 districts from eight divisions (Barisal, Chattogram, Dhaka, Khulna, Mymensingh, Rajshahi, Rangpur, and Sylhet) of Bangladesh were selected and described as strata assigning the entire population into residents of urban and rural areas (27). The MICS uses a two-stage stratified cluster sampling technique to determine the study sample. At first, census enumeration areas (CEA) within each stratum were selected to identify 3,220 primary sampling units. Then for the second sampling stage, 64,400 households were identified from the CEAs following selection of 20 homes in each sample area by systematic sampling (27). This survey assembled a variety of information using five validated questionnaires. The questionnaires were based on essential socio-demographic characteristics which were included in the household questionnaire, assessing the quality of water they use in the households, information of individual women aged 15–49 years, another one was for 5–17 years old children and an under-5 questionnaire, which was administered for the mothers or caregivers of those group of children. For this analysis, we used the household and under-5 questionnaires to extract data from the under-five (36–59 months) children, assessing the early childhood development index (Figure 1) (27).

### Outcome variable: early childhood development

ECD was estimated by the ECD index (ECDI), which has four domains: literacy- numeracy, learning, physical and social–emotional (2, 27). A sequence of questions was asked for each domain, and a certain number of positive responses was required to consider the child as having adequate development. The domain of literacy-numeracy was assessed by two of the three items: identifying at least ten letters, reading at least four famous words and names, and recognizing the symbol of all numbers from 1 to 10. The physical domain was measured by asking the mother or caregiver if the child is too sick to play sometimes and/or if the child can follow the simple direction correctly, like picking up a rock or stick from the ground with two fingers. If a child can follow directions properly and/or is not too sick to play sometimes, their physical domain is considered as developmentally on-track. The domain learning is comprised of two observations to indicate whether the child is on way with his/her learning domain. First, if the child can follow the simple direction correctly, and second when instruction is given, they are able to do it independently. The social–emotional domain was assessed to estimate emotional and social functioning and to appraise sensory processing. Three questions were collected from the mother or caregiver; if any of the two is true, the child is considered to be on-track. These are if the child gets along well with other children, does not kick, bite or hit other children, and does not get distracted easily. If a child met at least three of these four domains, the child was considered to be developmentally “on track,” and if any child who didn’t meet at least three domains was considered as developmentally “on delayed” (28). ECDI is then calculated as a percentage of children who are developmentally on-track in at least three of these four domains (27). We figured if a child was on track for each of the domains and labeled 0 as “delayed development” and 1 as “adequate development.” Overall, ECD was also determined and labeled in the same way.

### Primary explanatory variable: home environment

For home environment factors, we considered adult involvement in children’s activities, such as reading books or telling stories to children, singing songs, or playing with children (5, 29). The responses were recorded as whether the parents/caregivers/adults in the household participated in any of these activities. If parents/caregivers/adults in the household read at least a book to the child, then the variable was categorized as “no one reads a book” and someone reads a book” which were coded as 0 and 1, respectively. Similarly, all the home environment factors were categorized as 1 (otherwise 0) in this study if someone reads a book, tells a tale, sings a song, or plays with the child.

### Covariates

We adjusted a variety of child and caregiver factors as covariates. The covariates were broadly grouped as “children’s demographic factors,” maternal socioeconomic factors,” and “children’s nutritional status.” Children’s demographic factors included age of child and sex of the child, and administrative division. Children’s nutritional status included stunting and underweight. Stunting is a measure of linear growth and more specifically known as HAZ (height-for-age *Z* score), and underweight is a measure of weight-for-age *Z* score (WAZ). Children were categorized either as nourished (if *Z*-score ≥ −2.0), moderately stunted/ underweight (if −3.0 ≤ *Z*-score < −2.0), or severely stunted/ underweight (if *Z*-score < −3.0) according to WHO Child Growth Standards guideline (30). Maternal socioeconomic factors included the mother’s education, and household wealth index. The household wealth index was calculated using principal component analysis of the household characteristics and different household assets (27).

### Statistical analysis

This data analysis included summary statistics of distribution and cross-tabulation of indicators using R v3.6.1. For descriptive analysis, frequency and percentage were measured. This study included bivariate and multivariable models using binary logistic regression models to see the cofactors’ overall effects on the dependent variable. We also found cofactors on domain-wise development and overall ECD status in five separate models, respectively. The purpose of applying the models was to identify the factors contributing to the domains of ECD and its overall ECDI. The adjusted Odds Ratio (aOR) along with 95% confidence interval (CI) were used to interpret the findings. Statistical significance was set at 5% level (*p* < 0.05).

## Results

### Characteristics of the participants

The sample consisted of 7,326 children aged 3 to 4 years who lived in rural settings. The characteristics of the participants are given in Table 1. The majority of the participants (51.4%) were boys, and more than half were from middle-income families (61.6%), with 8.9% from high-income families. Nearly half of the enrolled children’s mothers (48.8%) finished their education up to the secondary level. Most participants (97.2%) came from homes with improved drinking water, and a comparable percentage from homes with improved toilet facilities. With 20.1% of the children having mild or moderate stunting, it was the most common form of anthropometric failure, followed by underweight (19.9%).

**Table 1:** Distribution of ECD status across different independent variables.

Variables	Total *N* (%)	Developmentally on track		Unadjusted OR (95% CI)	*p*-value
		Yes; *n* (%)	No; *n* (%)		
*Children’s demographic factors*					
Age of child (*n* = 7,326)					
3 years	3,684 (50.29)	2,440 (66.2)	1,244 (33.8)	1	<0.001
4 years	3,642 (49.71)	2,878 (79.0)	764 (21.0)	1.92 (1.73–2.13)	
Sex (*n* = 7,326)					
Boys	3,765 (51.39)	2,611 (69.3)	1,154 (30.7)	1	<0.001
Girls	3,561 (48.61)	2,707 (76.0)	854 (24.0)	1.40 (1.26–1.55)	
Administrative Division (*n* = 7,326)					
Dhaka	1,293 (17.65)	1,028 (79.5)	265 (20.5)	1	<0.001
Chattogram	1,438 (19.63)	1,084 (75.4)	354 (24.6)	0.79 (0.66–0.95)	
Barisal	675 (9.21)	448 (66.4)	227 (33.6)	0.51 (0.41–0.63)	
Khulna	1,045 (14.26)	718 (68.7)	327 (31.3)	0.57 (0.47–0.68)	
Mymensingh	460 (6.28)	279 (60.7)	181 (39.3)	0.39 (0.31–0.50)	
Rajshahi	837 (11.43)	584 (69.8)	253 (30.2)	0.59 (0.49–0.73)	
Rangpur	944 (12.89)	769 (81.5)	175 (18.5)	1.13 (0.92–1.40)	
Sylhet	634 (8.65)	408 (64.4)	226 (35.6)	0.46 (0.38–0.57)	
*Maternal socioeconomic factors*					
Wealth index (*n* = 7,326)					
Poor	2,163 (29.52)	1,481 (68.5)	682 (31.5)	1	<0.001
Middle	4,511 (61.58)	3,294 (73.0)	1,217 (27.0)	1.24 (1.11–1.39)	
Rich	652 (8.90)	543 (83.3)	109 (16.7)	2.29 (1.83–2.87)	
Mother’s education (*n* = 6,293)					
No education	1884 (29.94)	1,295 (68.7)	589 (31.3)	1	<0.001
Up to secondary	3,575 (56.81)	2,644 (74.0)	931 (26.)	1.29 (1.14–1.46)	
Higher secondary or above	834 (13.25)	671 (80.5)	163 (19.5)	1.87 (1.54–2.28)	
*Home environment factors*					
If anyone reads a book to the child (*n* = 7,323)					
No one reads a book	2,375 (32.43)	1,578 (66.4)	797 (33.6)	1	<0.001
Someone reads a book	4,948 (67.57)	3,739 (75.6)	1,209 (24.4)	1.56 (1.40–1.74)	
If anyone tells a story to the child (*n* = 7,324)					
No one tells a story	2,419 (33.03)	1,630 (67.4)	789 (32.6)	1	<0.001
Someone tells a story	4,905 (66.97)	3,687 (75.2)	1,218 (24.8)	1.46 (1.32–1.63)	
If anyone sings a song to the child (*n* = 7,325)					
No one sings a song	3,220 (43.96)	2,271 (70.5)	949 (29.5)	1	<0.001
Someone sings a song	4,105 (56.04)	3,047 (74.2)	1,058 (25.8)	1.20 (1.09–1.33)	
If anyone plays with the child (*n* = 7,326)					
No one plays with the child	3,047 (41.59)	2,193 (72.0)	854 (28.0)	1	0.317
Someone plays with the child	4,279 (58.41)	3,125 (73.0)	1,154 (27.0)	1.06 (0.95–1.17)	
*Children’s nutritional status*					
Stunting (*n* = 7,025)					
Severe stunting	577 (8.21)	374 (64.8)	203 (35.2%)	1	<0.001
Mild/ Moderate	1,470 (20.93)	1,011 (68.8)	459 (31.2)	1.19 (0.98–1.46)	
No stunting	4,978 (70.86)	3,733 (75.0)	1,245 (25.0)	1.63 (1.36–1.95)	
Underweight (*n* = 7,090)					
Severe underweight	369 (5.20)	234 (63.4)	135 (36.6)	1	<0.001
Mild/ Moderate	1,455 (20.52)	1,027 (70.6)	428 (29.)	1.38 (1.09–1.76)	
Healthy	5,266 (74.27)	3,900 (74.1)	1,366 (25.9)	1.65 (1.32–2.05)	

OR, Odds Ratio; CI, Confidence Interval.

### Prevalence of delayed ECD

About 27.4% children were identified as developmentally on delay, defined as not fulfilling at least three of the four domains of ECDI. When segregated by the domains, we found 72.7% of children missed the literacy-numeracy domain. In comparison, 1.8, 27.9, and 9.7% of the children did not attain physical, socio-emotional, and learning domains. According to the survey, around 32.4, 33, 44, and 41.6% of rural children did not have their parents who read books to them, tell them a story, sing a song to them, or play with them, respectively. The bivariate analysis showed that age and sex of the child, wealth index, mother’s education, nutritional status, and parental or caregivers’ interaction with children in their developmental activities were significantly associated with the ECD status (Table 1).

### Multivariable logistic regression models

Five different regression models were used for each domain of ECDI and overall ECDI to determine the factors associated with ECD status. Regarding the children’s demographic factors, children of the older age group (4 years) were more developmentally on-track in all the domains, with more than three times (aOR = 3.29, 95% CI = 2.90–3.74) higher odds in the literacy-numeracy domain and 2-fold higher (aOR = 2.02, 95% CI = 1.79–2.28) in case of overall ECDI compared to those of 3 years. In this study, girls were found to be higher developmentally on track in overall ECD status than boys (aOR = 1.39, 95% CI = 1.24–1.57). In division-wise comparison, children from most of the divisions except Rangpur had a significant delay in development than those from the Dhaka division. Children from the Mymensingh division were more likely to be developmentally on-track in the literacy-numeracy domain than those from the Dhaka division (Table 2).

**Table 2:** Multivariable logistic regression models provide adjusted odds ratio (95% Confidence interval) of each domain of adequate ECD.

Variables	Literacy numeracy	Physical	Social–emotional	Learning	Overall ECDI
	Adjusted Odds Ratio (95% Confidence Interval)				
*Age of children (Ref: 3 years)*					
Aged 4 years	3.29 (2.90–3.74)	1.60 (1.03–2.49)	1.28 (1.41–1.44)	1.48 (1.24–1.78)	2.02 (1.79–2.28)
Girls (Ref: Boys)	1.12 (0.99–1.26)	1.01 (0.66–1.55)	1.48 (1.32–1.67)	1.02 (0.86–1.22)	1.39 (1.24–1.57)
*Wealth index (Ref: Poor)*					
Middle	1.46 (1.24–1.71)	1.29 (0.79–2.12)	0.97 (0.84–1.12)	1.19 (0.96–1.47)	1.12 (0.97–1.29)
Rich	2.03 (1.59–2.59)	3.50 (0.98–12.49)	1.21 (0.95–1.55)	1.58 (1.07–2.31)	1.79 (1.37–2.34)
*Mother’s Education (Ref: No education)*					
Up to secondary	1.40 (1.20–1.63)	1.43 (0.87–2.35)	0.97 (0.84–1.11)	1.11 (0.90–1.36)	1.11 (0.97–1.28)
Higher secondary	2.04 (1.64–2.52)	0.70 (0.35–1.39)	1.06 (0.86–1.32)	1.21 (0.87–1.70)	1.39 (1.11–1.75)
*Home Environment (Ref: No)*					
Someone reads a book	3.63 (2.96–4.43)	0.49 (0.27–0.90)	1.02 (0.87–1.19)	1.21 (0.96–1.54)	1.26 (1.08–1.48)
Someone tells a story	1.34 (1.11–1.62)	1.93 (1.01–3.68)	1.09 (0.93–1.29)	0.88 (0.68–1.14)	1.29 (1.09–1.53)
Someone sings a song	1.15 (0.98–1.35)	0.82 (0.46–1.49)	1.03 (0.89–1.19)	0.98 (0.78–1.23)	1.06 (0.91–1.24)
Someone plays with the child	0.73 (0.63–0.84)	1.14 (0.68–1.93)	0.97 (0.85–1.11)	0.78 (0.63–1.27)	0.78 (0.68–1.49)
*Stunting status (Ref: Severe stunting)*					
No stunting	1.55 (1.13–2.12)	1.54 (0.67–3.49)	1.14 (0.88–1.47)	1.35 (0.95–1.92)	1.31 (1.02–1.68)
Mild/ Moderate	1.01 (0.73–1.39)	1.53 (0.66–3.53)	1.04 (0.80–1.34)	1.18 (0.84–1.68)	1.09 (0.85–1.41)
*Underweight status (Ref: Severe underweight)*					
Normal weight	1.35 (0.93–1.97)	1.47 (0.59–3.65)	1.14 (0.84–1.53)	1.29 (0.85–1.95)	1.32 (0.98–1.77)
Mild/Moderate	1.17 (0.80–1.71)	1.59 (0.63–3.96)	1.20 (0.88–1.60)	1.19 (0.79–1.79)	1.26 (0.94–1.69)
*Administrative Division (Ref: Dhaka)*					
Chattogram	1.03 (0.84–1.26)	0.71 (0.31–1.58)	0.56 (0.45–0.69)	0.74 (0.55–0.99)	0.79 (0.64–0.97)
Barisal	1.20 (0.94–1.52)	2.38 (0.64–8.83)	0.39 (0.30–0.49)	0.77 (0.51–1.03)	0.51 (0.39–0.64)
Khulna	0.72 (0.58–0.88)	1.49 (0.53–4.14)	0.37 (0.30–0.46)	1.09 (0.79–1.53)	0.52 (0.42–0.64)
Mymensingh	1.34 (1.01–1.80)	0.19 (0.08–0.41)	0.33 (0.25–0.44)	1.03 (0.66–1.62)	0.39 (0.30–0.52)
Rajshahi	0.66 (0.52–0.83)	0.98 (0.37–2.61)	0.52 (0.41–0.65)	1.15 (0.80–1.64)	0.56 (0.45–0.71)
Rangpur	0.91 (0.72–1.14)	0.32 (0.15–0.69)	0.99 (0.77–1.27)	1.23 (0.87–1.75)	1.14 (0.89–1.46)
Sylhet	1.01 (0.77–1.34)	1.35 (0.36–5.04)	0.45 (0.34–0.58)	0.61 (0.42–0.87)	0.53 (0.41–0.69)

When looking at the maternal socio-economic factors, mothers from households with rich wealth index was significantly associated with the ECD status. The children from affluent households had twice (aOR = 1.79, 95% CI = 1.37–2.34) more developmentally on track in overall ECDI than those from poor families, with 2.03 times (aOR = 2.03, 95% CI = 1.59–2.59) and 1.58times (aOR = 1.58, 95% CI = 1.07–2.31) more likely to attain literacy-numeracy and learning domains, respectively. Mother’s education was another significant factor of ECD for the literacy-numeracy domain and overall ECDI. The children of mothers having higher secondary education had 1.39 times (aOR = 1.39, 95% CI =1.11–1.75) higher odds of being developmentally on tract compared to those having no education. Similarly, children of mothers who had higher secondary or higher education revealed 2.04 times (aOR = 1.45, 95% CI = 1.22–1.74) higher odds of development in literacy-numeracy domain. Literacy numeracy and overall ECDI were found to be significantly associated with stunting. A healthy child showed 1.55 times higher literacy-numeracy attainment (aOR = 1.55, 95% CI = 1.13–2.12) and 1.39 times higher overall ECD status (aOR = 1.31, 95% CI = 1.02–1.68) compared to the children with severe stunting (Table 2).

The home environment factors, which include parental involvement in children’s activities, were also identified as significant associated with ECD status. For example, reading a book to the child had 3.63 times (aOR = 3.63, 95% CI = 2.96–4.43) and telling a story had 1.34 times (aOR = 1.34, 95% CI = 1.11–1.62) more likely to attain the literacy-numeracy compared to their counterparts. These activities are also significantly associated with their overall ECD status. Parents participating in telling stories to the child were 1.29 times (aOR = 1.29, 95% CI = 1.09–1.53) higher developmentally on track (Table 2).

## Discussion

According to this study, rural children aged 4–5 years without a supportive home environment were more likely to experience developmental delays. The age and sex of the child, household wealth index, and the mother’s level of education were significantly associated with the ECD status. Also, a substantial divisional variation has been revealed indicating some associations at the community level.

We found children of 4 years had a higher odd of having ECD on-track status than children of 3 years. This finding is also in line with the previous Bangladeshi studies worked on ECD status among children (28, 31). Age is also favorably correlated with the socio-emotional, literacy-numeracy, physical and learning domains, according to our research. Another study in Nepal found that older children had better development than younger children (2). Another study also identified that 4-year-old children had greater levels of cognitive and socio-emotional development than children who were 3 years old (14). This could be explained by taking into account the brain growth, where white matter volume rises linearly with age and has an impact on learning and motor functions of a child (32). In this study, the girls were more developmentally on-track than the boys, particularly in the social–emotional domain and overall ECD status. A similar finding was found where boys showed delayed development than girls (1, 28, 33). Emerson et al. also showed that Bangladesh, Pakistan, and Vietnam had higher rates of males with developmental delays than females (32).

After accounting for other variables, mother’s higher education was found to be a significant factor associated with the literacy-numeracy domain, and overall ECDI. This result supports the findings of previous studies that lower education of mothers is significantly associated with the delayed development outcomes for children (1, 28, 34). As the early life of a child is nurtured by their mother exclusively, educated mothers have better communication and learning skills, which in turn influences better development (35). Furthermore, a plausible explanation of our finding could be that a mother’s education is crucial for a child’s early growth, and her intellect has an impact on the child’s development as they grow older (36, 37).

Compared to the poor counterpart, children from households with rich wealth status had better chance to attain overall ECD status, particularly in the literacy-numeracy and learning domains. This result aligns with the previous similar Bangladeshi studies (28, 31). This is due to that low socio-economic condition is associated with adequate food, safe water, and sanitation facilities as well as a mother’s education which in dearth provokes inadequate development and increases susceptibility to infections (34). Previous study also identified an association between lower wealth index and poor development of children (38). A study in Kenya revealed that inadequate family income negatively affects ECD (35). The aforementioned problems may frequently appear together, and their combined effect has a negative impact on a child’s overall development, which can be seen in infancy but worsens over time. A study also discovered that children from low-income households could not develop the same level of cognitive and verbal skills as children from higher-income families (17).

When looking at the home environment factors, we found that reading books and telling stories to the children were positively associated with the overall ECD status and literacy-numeracy domain. Previous literature also documented that childhood growth had a significant relationship with parental involvement in children’s learning activities such as reading books, and telling stories to them (28, 31, 39). A Bangladeshi study also identified that if a child has access to children’s books and their mother or other primary caregiver reads or tells stories to them, the child responds favorably to the development of the literacy-numeracy domain (31). The house and its surroundings are critical for a child’s early development since the home and its surroundings provide the child with their first lesson, care, and nourishment (7). The influence of the home environment was revealed to be a significant factor on cognitive development (40, 41). It is evidenced that the development of a child’s motor function is greatly influenced by the home environment and parent–child interactions (42).

Stunting was negatively associated with the ECD status in this study. Early stunting is caused by undernutrition and recurrent infection, both of which could be results of poor wealth quintile (43, 44). The literacy-numeracy domain and overall development ECDI were found to be adversely impacted by severe stunting. Children with any kind of stunting were related to significantly lower ECD scores in a prior study conducted among 98,189 children aged 36–59 months in 34 Low-and-Middle-Income Countries (LMICs) (3). Stunted children also showed significantly lower scores of ECD than their healthy peers on cognitive, motor, language, and social–emotional scales in a MAL-ED study in Bangladesh (34).

### Strengths and limitations

The study gives new epidemiological evidence that could be used to do more research on the effects of a supportive home environment on ECD. Still, using these measures gave us useful information about how different home environment variables are related to ECD. The best thing about this study is that it is a nationwide survey with a large sample of 36- to 59-month-old children. This is based on the fact that rural people are often ignored, which makes the study stronger. It was a cross-sectional study that limits causal inference. Moreover, some information regarding ECD was retrospective, which may introduce recall bias. Another limitation of this study is the age group to investigate ECD, which was confined to three to 4 years, which limits us from evaluating the changes that occur after 4 years of children. The MICS survey data are limited to its available covariates. Therefore, it was impossible to control for some essential factors like the nutritional status of parents, genetic factors, and children’s dietary intake, which could be significant contributors to ECD. Besides, we only incorporated data from rural children only to know the scenario of the ECD status in the rural context and to know its association with home environment factors; however, disaggregated data by urban–rural areas would be instrumental in understanding the impact of home environment on ECD among overall Bangladeshi children.

## Conclusion

Home environment factors like reading books and telling stories to children were found to be significantly associated with ECD in rural areas of Bangladesh. Besides, most rural Bangladeshi children who were developmentally on-track belonged to households with higher wealth status than those from poor households, who typically had developmental delays. Delayed childhood development was higher among children of mothers with little or no formal education. Our study’s findings would assist in implementing the essential public health intervention focusing on the targeted factors to enhance the ECD program especially in the rural Bangladeshi context. Hence, policymakers and public health professionals working to enhance ECD status should consider the study’s findings. Policymakers in rural Bangladesh may also emphasize enhancing mother’s education and training to enrich home environments through cognitive development to enhance ECD status.

## Data availability statement

Publicly available datasets were analyzed in this study. This data can be found at: https://mics.unicef.org/surveys.

## Ethics statement

Ethical review and approval was not required for the study on human participants in accordance with the local legislation and institutional requirements. Written informed consent to participate in this study was provided by the participants’ legal guardian/next of kin.

## Author contributions

FR and SNT: conceptualization, methodology, data curation, formal analysis, writing–original draft. PM, SS, AH, and SK: Writing–original draft, and review and editing. AAC and AH: conceptualization, methodology, data curation, formal analysis, writing–original draft, review and editing, and supervision. All authors read and approved the final manuscript.

## Conflict of interest

The authors declare that the research was conducted in the absence of any commercial or financial relationships that could be construed as a potential conflict of interest.

## Publisher’s note

All claims expressed in this article are solely those of the authors and do not necessarily represent those of their affiliated organizations, or those of the publisher, the editors and the reviewers. Any product that may be evaluated in this article, or claim that may be made by its manufacturer, is not guaranteed or endorsed by the publisher.

## Abbreviations

ECD, Early childhood development
ECDI, Early childhood development index
LMICs, low and middle-income countries
SDG, Sustainable development Goal
MICS, Multiple Indicator Cluster Survey
CIAF, composite index of anthropometric failure
CEA, census enumeration areas
PSU, Primary sampling unit
OR, Odds ratio
CI, Confidence interval
LN, Literacy-numeracy.

## References

[1] Alam MI Mansur M Barman P. Early childhood development in Bangladesh and its socio-demographic determinants of importance. Early Child Dev Care. (2022) 192:1901–920. doi: 10.1080/03004430.2021.1951260

[2] Sk R Banerjee A Mishra R Barua S. Quality of care and early childhood developmental status in Nepal: a multilevel analysis. Early Child Dev Care. (2019) 190:2264–77. doi: 10.1080/03004430.2019.1570503

[3] Bornstein MH Rothenberg WA Lansford JE Bradley RH Deater-Deckard K Bizzego A. Child development in low-and middle-income countries. Pediatrics. (2021) 148:e2021053180. doi: 10.1542/peds.2021-053180 34642232

[4] United Nations. Sustainable development goals (2015). Available at: https://www.un.org/sustainabledevelopment/education/ (accessed June 1, 2023).

[5] Black MM Walker SP Fernald LCH Andersen CT DiGirolamo AM Lu C. Early childhood development coming of age: science through the life course. Lancet. (2017) 389:77–90. doi: 10.1016/S0140-6736(16)31389-7, PMID: 27717614 PMC5884058

[6] Leventhal T Brooks-Gunn J. Poverty and child development In: Smelser NJ, Baltes PBBT-IE of the S& BS, editors. International encyclopedia of the Social & Behavioral Sciences. Oxford: Pergamon (2001)

[7] Molfese VJ Modglin A Molfese DL. The role of environment in the development of reading skills: a longitudinal study of preschool and school-age measures. J Learn Disabil. (2003) 36:59–67. doi: 10.1177/00222194030360010701 15490892

[8] Bradley R Corwyn R. Caring for children around the world: a view from HOME. Int J Behav Dev. (2005) 29:468–8. doi: 10.1177/01650250500146925

[9] Andrade SA Santos DN Bastos AC Pedromônico MRM Almeida-Filho N de Barreto ML. Family environment and child’s cognitive development: an epidemiological approach. Rev Saude Publica (2005) 39:606–1, doi: 10.1590/S0034-89102005000400014, PMID: 16113911

[10] Prado EL Dewey KG. Nutrition and brain development in early life. Nutr Rev. (2014) 72:267–4. doi: 10.1111/nure.12102 24684384

[11] Bradley RH Whiteside L Mundfrom DJ Casey PH Kelleher KJ Pope SK. Early indications of resilience and their relation to experiences in the home environments of low birthweight, premature children living in poverty. Child Dev. (1994) 65:346–09. doi: 10.2307/1131388 8013226

[12] Sultana ZZ Arefin A Hossain A. Addressing child protection issues in Bangladesh's Rohingya and host community to improve children's health. Lancet Reg Health Southeast Asia. (2022) 5:100070. doi: 10.1016/j.lansea.2022.100070 37383670 PMC10306008

[13] Shonkoff JP Garner AS Health C on PA of C and F. The lifelong effects of early childhood adversity and toxic stress. Pediatrics. (2012) 129:e232–46. doi: 10.1542/peds.2011-2663, PMID: 22201156

[14] McCoy DC Peet ED Ezzati M Danaei G Black MM Sudfeld CR. Early childhood developmental status in low-and middle-income countries: national, regional, and global prevalence estimates using predictive modeling. PLoS Med. (2016) 13:e1002034. doi: 10.1371/journal.pmed.1002034, PMID: 27270467 PMC4896459

[15] UNICEF. The formative years: UNICEF‘s work on measuring early childhood development [internet]. New York: UNICEF (2014).

[16] Hossain MS Siddiqee MH Ferdous S Faruki M Jahan R Shahik SM. Is childhood overweight/obesity perceived as a health problem by mothers of preschool aged children in Bangladesh? A community level cross-sectional study. Int J Environ Res Public Health. (2019) 16:202. doi: 10.3390/ijerph16020202 30642056 PMC6352241

[17] Roopnarine JL Dede YE. Paternal and maternal engagement in play, story telling, and reading in five Caribbean countries: associations with preschoolers’ literacy skills. Int J Play. (2018) 7:132–5. doi: 10.1080/21594937.2018.1496000

[18] Frongillo EA Kulkarni S Basnet S de Castro F. Family care behaviors and early childhood development in low-and middle-income countries. J Child Fam Stud. (2017) 26:3036–44. doi: 10.1007/s10826-017-0816-3

[19] Muñez D Bull R Lee K. Maternal education and siblings: agents of cognitive development in kindergarten. Dev Sci. (2021):e13218. doi: 10.1111/desc.13218 34964196

[20] Kar BR Rao SL Chandramouli BA. Cognitive development in children with chronic protein energy malnutrition. Behav Brain Funct. (2008) 4:31–12. doi: 10.1186/1744-9081-4-31 18652660 PMC2519065

[21] Dewey KG Begum K. Long-term consequences of stunting in early life. Matern Child Nutr. (2011) 7:5–18. doi: 10.1111/j.1740-8709.2011.00349.x, PMID: 21929633 PMC6860846

[22] Islam M Sultana ZZ Iqbal A Ali M Hossain A. Effect of in-house crowding on childhood hospital admissions for acute respiratory infection: a matched case-control study in Bangladesh. Int J Infect Dis. (2021) 105:639–5. doi: 10.1016/j.ijid.2021.03.002, PMID: 33684561

[23] McGrath M. Advancing early childhood development: from science to scale. F Exch. (2016) 53:41.

[24] Irwin LG Siddiqi A Hertzman G. Early child development: a powerful equalizer. Citeseer (2007).

[25] Kundu S Das P Rahman MA Al Banna MH Fatema K Islam MA. Socio-economic inequalities in minimum dietary diversity among Bangladeshi children aged 6-23 months: a decomposition analysis. Sci Rep. (2022) 12:21712. doi: 10.1038/s41598-022-26305-9, PMID: 36522494 PMC9755277

[26] UNICEF Bangladesh. Baseline survey of caregivers kap on early childhood development in Bangladesh, vol. 1. Dhaka: UNICEF Bangladesh (2001).

[27] Bangladesh Bureau of Statistics (BBS) and UNICEF Bangladesh. Progotir Pathey, Bangladesh Multiple Indicator Cluster Survey (2019). Survey findings report. Dhaka: Bangladesh: Bangladesh Bureau of Statistics (BBS) (2019.

[28] Hasan MN Babu MR Chowdhury MAB Rahman MM Hasan N Kabir R. Early childhood developmental status and its associated factors in Bangladesh: a comparison of two consecutive nationally representative surveys. BMC Public Health. (2023) 23:1–13. doi: 10.1186/s12889-023-15617-8 37046226 PMC10099688

[29] Britto PR Lye SJ Proulx K Yousafzai AK Matthews SG Vaivada T. Nurturing care: promoting early childhood development. Lancet. (2017) 389:91–2. doi: 10.1016/S0140-6736(16)31390-3 27717615

[30] WHO Multicentre Growth Reference Study. WHO child growth standards based on length/height, weight and age. Acta Paediatr. (2006) 95:76–85. doi: 10.1111/j.1651-2227.2006.tb02378.x

[31] Alam MI Mansur M Barman P. Early childhood development in Bangladesh and its socio-demographic determinants of importance. Early Child Dev Care. (2022) 192:1901–20. doi: 10.1080/03004430.2021.1951260

[32] Fields RD. White matter in learning, cognition and psychiatric disorders. Trends Neurosci. (2008) 31:361–09. doi: 10.1016/j.tins.2008.04.001 18538868 PMC2486416

[33] Haq I Hossain M Zinnia MA Hasan MR Chowdhury I-A-Q. Determinants of the early childhood development index among children aged< 5 years in Bangladesh, Costa Rica and Ghana: a comparative study. East Mediterr Heal J. (2021) 27:1069–77. doi: 10.26719/emhj.21.055 34927710

[34] Nahar B Hossain M Mahfuz M Islam MM Hossain MI Murray-Kolb LE. Early childhood development and stunting: findings from the MAL-ED birth cohort study in Bangladesh. Matern Child Nutr. (2020) 16:e12864. doi: 10.1111/mcn.12864 31237738 PMC7038907

[35] Ong’ayi DMM Dede Yildirim E Roopnarine JL. Fathers’, mothers’, and other household members’ involvement in reading, storytelling, and play and preschoolers’ literacy skills in Kenya. Early Educ Dev. (2020) 31:442–4. doi: 10.1080/10409289.2019.1669125

[36] Curenton SM Justice LM. Children’s preliteracy skills: influence of mothers’ education and beliefs about shared-reading interactions. Early Educ Dev. (2008) 19:261–3. doi: 10.1080/10409280801963939

[37] Schady N. Parents’ education, mothers’ vocabulary, and cognitive development in early childhood: longitudinal evidence from Ecuador. Am J Public Health. (2011) 101:2299–07. doi: 10.2105/AJPH.2011.300253, PMID: 22021308 PMC3222428

[38] Tran TD Luchters S Fisher J. Early childhood development: impact of national human development, family poverty, parenting practices and access to early childhood education. Child Care Health Dev. (2017) 43:415–6. doi: 10.1111/cch.12395, PMID: 27535624

[39] Wang B Luo X Yue A Tang L Shi Y. Family environment in rural China and the link with early childhood development. Early Child Dev Care. (2022) 192:617–09. doi: 10.1080/03004430.2020.1784890

[40] Biedinger N. The influence of education and home environment on the cognitive outcomes of preschool children in Germany. Child Dev Res. (2011) 2011:1–10. doi: 10.1155/2011/916303

[41] Moore TG McDonald M Carlon L O’Rourke K. Early childhood development and the social determinants of health inequities. Health Promot Int. (2015) 30:ii102–15. doi: 10.1093/heapro/dav031 26420806

[42] Saccani R Valentini NC Pereira KRG Müller AB Gabbard C. Associations of biological factors and affordances in the home with infant motor development. Pediatr Int. (2013) 55:197–3. doi: 10.1111/ped.12042, PMID: 23279095

[43] Baye K Laillou A Chitweke S. Socio-economic inequalities in child stunting reduction in sub-Saharan Africa. Nutrients. (2020) 12:253. doi: 10.3390/nu12010253, PMID: 31963768 PMC7019538

[44] Angdembe MR Dulal BP Bhattarai K Karn S. Trends and predictors of inequality in childhood stunting in Nepal from 1996 to 2016. Int J Equity Health. (2019) 18:1–17. doi: 10.1186/s12939-019-0944-z 30836975 PMC6402091

